# Adeno-Associated Virus-Engineered Umbilical Cord-Derived Mesenchymal Stromal Cells Overexpressing Human sFlt-1 for Anti-Angiogenesis

**DOI:** 10.3390/life15050728

**Published:** 2025-04-30

**Authors:** Ewa Yee-Wa Choy, Chee-Onn Leong, Soon-Keng Cheong, Khong-Lek Then, Kong-Yong Then

**Affiliations:** 1 School of Postgraduate Studies, Faculty of Medicine and Health, IMU University, Jalan Jalil Perkasa 19, Bukit Jalil, Kuala Lumpur 57000, Malaysia; ewa@cryocord.com.my; 2 CryoCord Sdn Bhd, 1, Bio X Centre, Persiaran Cyber Point Selatan, Cyberjaya 63000, Selangor, Malaysia; 3 AGTC Genomics, J2-1, Pusat Perdagangan Bandar, Persiaran Jalil 1, Kuala Lumpur 57000, Malaysia; 4 Hematology Department, National University of Malaysia (UKM), Jalan Ya’acob Latif, Bandar Tun Razak, Cheras, Kuala Lumpur 56000, Malaysia; 5 Department of Medicine, M. Kandiah Faculty of Medicine and Health Sciences, Universiti Tunku Abdul Rahman, Jalan Sungai Long, Bandar Sungai Long Cheras, Kajang 43000, Selangor, Malaysia

**Keywords:** mesenchymal stromal cells, adeno-associated virus, genetic engineering, anti-angiogenesis

## Abstract

Purpose. Genetic engineering of mesenchymal stromal cells (MSCs) using viral vectors has emerged as a promising approach to enhance the efficacy of anti-angiogenic gene therapies. Umbilical cord-derived MSCs are an attractive cell source due to their easy accessibility and potential for genetic modification. Adeno-associated viruses (AAVs) have been utilized in clinical settings to deliver therapeutic genes due to its characteristic of transient integration into the genome. In this study, we investigated the efficacy of using recombinant AAV-engineered umbilical cord-derived MSCs overexpressing anti-angiogenic factor, hsFlt-1 (MSCs.hsFlt1). Methods. The plasmid containing the hsFlt-1 gene was cloned into the AAV2 target backbone and validated using Sanger sequencing. The transduction process was studied to determine the optimal conditions, including the effect of MOI, media serum percentage, and attachment of MSCs, to achieve higher transduction efficiency. The functionality of MSCs.hsFtl1 was analyzed using qPCR, ELISA, and tube formation assays. Results. MSCs.hsFtl1 transduced at an MOI of 1 × 10^6^ demonstrated high transduction efficiency and exhibited robust gene and protein expression of hsFlt-1. The results revealed significant inhibition of growth in human umbilical vein endothelial cells (HUVECs) using a remarkably low dose of MSCs.hsFlt1 at 12.3 ng/mL. This observed anti-angiogenic effect was comparable to the clinically used Bevacizumab. Conclusions. The anti-angiogenic potential of MSCs.hsFlt1 effectively demonstrated in this study suggests their promising utility for targeted anti-angiogenic gene therapy approaches.

## 1. Introduction

Recent advancements have led to the use of genetic engineering in anti-angiogenesis with various gene delivery vehicles. Friedenstein et al. first discovered mesenchymal stromal cells (MSCs) in 1976; since then, cell-based therapy using MSCs has been extensively studied in the field of regenerative medicine and tissue engineering [1]. MSCs can be an excellent choice of delivery vehicles due to their relative ease of isolation from various human tissues, such as Wharton’s jelly from the umbilical cord, adipose tissue, and dental tooth pulp. The genetic engineering of MSCs using viral vector systems has emerged as a promising strategy to enhance their therapeutic potential for various applications, including tumor cell inhibition, bone regeneration, and anti-angiogenic therapy [2,3]. Wen et al. explored the use of genetically modified allogeneic bone marrow-derived MSCs by overexpressing the hepatocyte growth factor (HGF) gene using adenoviral vectors [4]. Transplantation of HGF-transgenic MSCs was performed one week after traumatic osteonecrosis of the femoral head in a rabbit model. The results showed recovery with decreased empty lacunae and increased VEGF expression. Another group of authors modified bone marrow-derived MSCs using AAV to carry the pigment epithelial-derived factor (PEDF) gene, to inhibit tumor angiogenesis, leading to apoptosis in gliomas [5]. A total of 1 × 10^6^ modified MSCs were injected into the tail vein of mice every two days for the duration of the experiment, for 40 days. The results showed that the survival times of the mice injected with modified MSCs were significantly longer than those injected with unmodified MSCs. Most cellular-based genetic modification studies have predominantly used bone marrow-derived MSCs, largely due to their early discovery. However, the increasing use of other sources, such as WJ-MSCs, provides insights that these sources may offer a better profile for therapeutic indications [6]. MSCs derived from umbilical cord tissue have especially gained significant attention in the field of regenerative medicine due to their multipotent differentiation capacity, homing to injury sites, and immunomodulatory properties [7]. Umbilical cords are often discarded as medical waste and are thus easily available, and the collection procedure is non-invasive. MSCs derived from Wharton’s jelly (WJ-MSCs) extracted from the umbilical cord can be expanded to a large degree with a higher doubling time compared to adipose tissue, thus making WJ-MSCs a feasible source for treatment [8].

Previous works on over-expressing anti-angiogenic genes using a non-viral vector showed relatively low efficiency and short-term expression [9,10]. Other studies have demonstrated that gene transduction is more efficient in a viral system than in a non-viral system [11]. However, the viral delivery system is often associated with immunogenicity that might lead to toxicity and insertional mutagenesis. Viral vectors such as adeno-associated viruses (AAVs) are increasingly favored for in vitro and in vivo gene transfer due to their high transduction efficiency, broad tissue tropism, and proven safety profiles [12]. These characteristics make AAV vectors particularly suitable for engineering cells to overexpress therapeutic proteins.

VEGF plays a pivotal role in the pathogenesis of several diseases related to aberrant angiogenesis, including various ocular disorders and conditions characterized by neovascularization. Understanding the contribution of VEGF in the formation of new vessels is fundamental to the development of effective anti-VEGF therapies. Research has demonstrated that VEGF initiates and sustains pathological neovascularization, and its inhibition via VEGF receptor 1 (VEGFR-1 and Flt) or VEGF receptor 2 (VEGFR-2, Flk, and KDR) signaling effectively curtails neovascularization and tumor growth [13].

The soluble form of VEGFR-1, known as soluble fms-like tyrosine kinase-1 (sFlt-1), has emerged as a potent VEGF antagonist with significant implications for angiogenesis regulation [14]. As an alternatively spliced variant, sFlt1 includes only the first six extracellular immunoglobulin (Ig)-like domains, lacking the transmembrane and intracellular tyrosine kinase domains of the full-length VEGFR-1 [15]. This truncated structure allows sFlt1 to effectively inhibit VEGF by preventing its interaction with membrane-bound VEGF receptors, thereby disrupting VEGF signaling pathways and impeding angiogenic processes.

In this study, we explore the potential of AAV-engineered umbilical cord-derived MSCs overexpressing sFlt1 as an anti-angiogenic gene therapy. By harnessing the capabilities of AAV vectors and the therapeutic potential of sFlt-1, we aim to develop a robust and effective treatment modality for angiogenesis-related diseases. To test our hypothesis, we conducted in vitro assays using endothelial cells to assess the anti-angiogenic effects of these engineered MSCs. This innovative approach promises to enhance our understanding of anti-angiogenic gene therapy and offers a potent strategy for treating diseases associated with pathological angiogenesis.

## 2. Materials and Methods

### 2.1. Construction of Plasmids

The kit of the AAV helper-free expression system was employed to generate AAV particles without the need for a helper virus (Cat #6230; Takara Bio USA, Inc., San Jose, CA, USA). The plasmid encoding cDNA of the gene of interest (GOI), specifically the human FLT1 gene soluble fragment (7 Ig-like domains), was separately obtained for cloning (Cat #puno1-hflt1s; InvivoGen, San Diego, CA, USA). The In-Fusion HD Cloning Kit (Cat #638909; Takara, USA) was utilized for precise cloning. The process began by using the In-Fusion online software (Takara Bio USA, Inc., San Jose, CA, USA) to simulate and determine the specific fragment to be cut and cloned from the GOI plasmid into the AAV helper-free plasmid (pAAV.hFLT1s7). A pair of Polymerase Chain Reaction (PCR) primers was designed for single-insert cloning into the linearized AAV plasmid: Forward primer: 5′-AAG AAT TGG GAT TCG CGA CAT GGT CAG CTA CTG GGA CAC C-3′ and Reverse primer: 3′-TGC CAC CCG TGG ATC CCG AAT TCT ACA CAG AGC CCT TC-5′. These primers were used to amplify the GOI fragment using the PCR method. The PCR product was analyzed on a 1% agarose gel and purified using the NucleoSpin Gel and PCR Clean-up Kit (Promega Corporation, Madison, WI, USA). The AAV helper-free plasmid was linearized using two restriction enzymes: Nru I (Cat #R7091; Promega Corporation, Madison, WI, USA) and BamH I (Cat #R6021; Promega Corporation, Madison, WI, USA) based on the manufacturer’s protocol. The reaction was incubated at 37 °C for 1 h. Post-digestion, the linearized plasmid was purified using the NucleoSpin Gel and PCR Clean-up Kit. The purified PCR product (GOI) and linearized AAV plasmid were combined in the In-Fusion cloning reaction (Cat #638909; Takara Bio USA, Inc., San Jose, CA, USA). The In-Fusion reaction mixture was transformed with chemically competent E. coli (Stellar Competent Cells, Cat #636763; Takara Bio USA, Inc., San Jose, CA, USA). Colonies were screened using colony PCR, and positive clones were confirmed by restriction enzyme analysis and Sanger sequencing (Macrogen Asia Pacific, Singapore). The results were analysed using the molecular biology research software SnapGene version 5.2.4 (GSL Biotech LLC., San Diego, CA, USA).

The enhanced green fluorescent protein fragment (eGFP) was amplified using the AAV.hPDGFRA.eGFP plasmid (a generous gift from Edward Callaway, Addgene plasmid #22919, Watertown, MA, USA). To maintain reliability across the experiment, the same AAV helper-free plasmid backbone was used. A pair of primers was specifically designed to linearize the plasmid and facilitate the ligation of the eGFP insert: Forward Primer: 5′-AAG AAT TGG GAT TCG ATG GTG AGC AAG GGC GAG G-3′ and Reverse Primer: 3′-TGC CAC CCG TGG ATC CTT GTA CAG CTC GTC CAT GCC-5′. This approach ensured accurate integration of the eGFP gene into the AAV plasmid (pAAV.eGFP), allowing for subsequent validation and expression analysis.

### 2.2. Recombinant AAV Production

The plasmids produced were packaged using the HEK293T cell line (Takara, USA) to produce recombinant AAV (rAAV) viral particles. The complete growth medium containing Dulbecco’s Modified Eagle’s Medium (DMEM) with high glucose (4.5 g/L) (Sigma-Aldrich, St. Louis, MO, USA), 10% Fetal Bovine Serum (FBS; Gibco, Grand Island, NY, USA), and 1% Penicillin G Sodium-Streptomycin sulfate (PenStrep; Gibco) was used to culture and expand HEK293T cells. The cells were co-transfected with pAAV.hFLT1s7, pAAV.RC2 and pAAV.Helper plasmids using the calcium phosphate method. The same production process for viral particles was repeated by replacing the target plasmid with pAAV.eGFP. The transfected cells were incubated overnight at 37 °C with 5% CO_2_. The medium was replaced the next day with fresh DMEM medium containing high glucose, 2% FBS, and 1% PenStrep. The transfected cells were collected 72 h post-transfection by adding AAV-MAX Lysis Buffer (Cat #A50520; Gibco) and 90 U/mL Benzonase nuclease (Sigma-Aldrich, St. Louis, MO, USA) directly to the culture flasks and incubated at 37 °C for 2 h on an orbital shaker. The rAAV crude lysate was transferred to a centrifuge tube and centrifuged at 4500× *g* at 4 °C for 30 min. The supernatant containing rAAV crude lysate, which is the primary virus stock, was transferred to a new tube. The rAAV was purified using iodixanol density in ultracentrifugation using a Ti70 rotor (Beckman Coulter, Indianapolis, IN, USA) for 1 h at 461,300× *g* (equivalent to 66,900 rpm) at 18 °C. The purified viral particles were collected using 21-G needles after the gradient steps. The purified rAAV viral particles were subject to a concentration and buffer exchange protocol, which was modified from Crosson et al. [16]. The purified concentrated rAAV viral particles were treated with DNase I and incubated at 37 °C for 15 min. DNase I-treated concentrated rAAV particle titrated using a Quantitative PCR (qPCR) machine (Applied Biosystems, ABI; Thermo Fisher Scientific, Foster City, CA, USA).

### 2.3. Transduction of MSCs

Six biological donor MSC cell lines derived from human umbilical cord tissue were obtained for this study with informed consent (a generous gift provided by CryoCord Sdn Bhd, Malaysia). The cells were cultured and maintained in a proprietary complete growth medium comprising 5% platelet lysate complete media (PLCM) with Dulbecco’s Modified Eagle’s Medium (DMEM) F12, also supplied by CryoCord Sdn Bhd. Donor MSCs at passage 3 were seeded in 96-well plates at a seeding density of 4000 cells per well and incubated overnight at 37 °C and 5% CO_2_. The following day, the cells were transduced with concentrated rAAV viral particles encoding hFLT1(s7) at a multiplicity of infection (MOI) ranging from 1 to 1 × 10^6^ in triplicate. After 16 to 18 h of incubation, the media were replaced and the cells were cultured for an additional three days. Transduced MSCs were subsequently harvested for downstream analyses to evaluate anti-angiogenic effects, including assessments of gene expression, protein expression, and endothelial functional assay.

### 2.4. Gene Expression with qPCR

The primers used in this study were designed using Primer 3 version 4.1.0 software (https://primer3.ut.ee/ accessed on 6 March 2021). The primary sequences are obtained from the NCBI Gene Bank database (http://blast.ncbi.nlm.nih.gov/Blast.cgi accessed on 6 March 2021). Table 1 shows the genes for qPCR analysis. The total ribonucleic acid (RNA) of all samples is extracted manually using TRI reagents with the addition of 200 μL of chloroform into each sample tube as previously described [17]. The cDNA was synthesized using SuperScript^®^ III First-Strand Synthesis SuperMix for qRT-PCR kit (Cat #18080-400; Invitrogen, Waltham, MA, USA) based on the manufacturer’s protocol. Master Mix containing SYBR^®^ Select Master Mix (Cat #4472908; Applied Biosystems, ABI; Thermo Fisher Scientific, Foster City, CA, USA), cDNA templates, and RNase and DNase-free water were assembled. The qPCR cycles were performed using an ABI thermal cycler machine.

### 2.5. Protein Quantification with ELISA

An enzyme-linked immunosorbent assay (ELISA) was performed to quantify the protein expression of hFLT1(s7) based on the manufacturer’s protocol (Cat #BMS268-3; Invitrogen, Thermo Fisher Scientific, Waltham, MA, USA). A sandwich ELISA was used for higher efficiency in detecting sample antigens. The absorbances were measured using a spectrophotometer at 450 nm and 620 nm as the reference wavelengths. The standard curve was plotted, and the concentrations of hFLT1(s7) for all the samples were calculated.

### 2.6. Tube Formation Assay

The tube formation assay was performed using a co-culture system. Transduced MSCs were pre-seeded on a hanging transwell insert (Cat #35024; SPL, Seoul, Republic of Korea) with a pore size of 0.4 μm and cultured for three days prior to the assay. The inserts were later transferred to 24-well plates pre-coated with Human Umbilical Vein Endothelial Cells (HUVECs; American Type Culture Collection (ATCC), Manassas, VA, USA) and Matrigel Growth Factor Reduced Basement Membrane Matrix (Cat #354230; Corning, Merck, Corning, NY, USA). HUVECs were cultured using an angiogenesis starter kit comprising Human Large Vessel Endothelial Cell Basal Medium (HLVE; Cat #A1460901; Gibco, Thermo Fisher, Grand Island, NY, USA) supplemented with a low serum growth supplement kit. For the control group, Bevacizumab (Avastin; Genentech, South San Francisco, CA USA) was included in the experiment.

On the day of the assay, chilled Matrigel was added to the pre-chilled 24-well plate on ice and incubated at 37 °C for 1 h to polymerize. Simultaneously, HUVECs were trypsinized, resuspended in HLVE basal medium at a concentration of 1.2 × 10^5^ cells/well, seeded onto the coated plates, and incubated at 37 °C with 5% CO_2_ for three hours to allow tube formation. The MSC-seeded transwell inserts were then transferred to the assay plates and incubated for an additional 16 h. Following this incubation, transwell inserts were removed, and 300 µL/well of Calcein AM (Corning, Merck, Corning, NY, USA) labeling solution was added and incubated for 1 h. Afterward, the labeling solution was removed, and the wells were washed twice with Hanks’ Balanced Salt Solution (HBSS; Gibco, Thermo Fisher, Grand Island, NY, USA). Fluorescent images of the formed tubes were captured using a microscope and analyzed with WimTube software (Wimasis, Onimagin Technologies SCA, Nuremberg, Bavaria, Germany).

### 2.7. Statistical Analysis

The data were analyzed using Student’s *T*-test to identify significance at *p* < 0.05, whereas, when samples were not equal, a one-way ANOVA test was performed to study the significance of results at *p* < 0.05 and followed by post-hoc analysis using Student’s *T*-test.

## 3. Results

### 3.1. Analysis and Validation of Successful Cloning

The specific region containing hsFlt1 sequences from the original vector, UNO1.hFlt1(s7), was amplified with conventional PCR. This specific PCR product with 2255 base pairs (bp) was confirmed by gel electrophoresis on 1% agarose gel as a single band and showed bands lightly above 2 kb, which is consistent with 2255 bp of the hsFlt1 plasmid. All the purified fragments, including hsFlt1 and eGFP, were sub-cloned and ligated into the backbone vector, pAAV.CMV. Restriction enzymes, NRU I and BamHI, were used to digest and clone this region. These successfully ligated new plasmids were identified as AAV.hsFlt1 and AAV.eGFP, whereas pAAV.CMV without cloning in any gene of interest (GOI) was relabeled as AAV.Ori to serve as a positive control throughout the study. Colony PCR was performed to identify successful individual transformants with the presence of ligated plasmids constructs of AAV.hsFlt1 and AAV.eGFP.

Successful cloning of each ligated plasmid was analysed using Sanger Sequencing. The alignment of full sequences for AAV.hsFlt1 and AAV.eGFP was performed using the molecular biology software SnapGene version 5.2.4 (San Diego, CA, USA). A set of primers was designed to walk the full sequences of both ligated plasmids, respectively. Sanger sequencing results demonstrated complete consistency in the inserted fragment with the exact size and sequences of hsFlt1 and eGFP nucleotide acids.

### 3.2. Production of rAAV Viral Particles

The concentrations of each plasmid were measured, and the purity of DNA was represented by A260/A280, with a purity ratio range of 1.8 to 2.0, which is generally acceptable for downstream applications. Only plasmids with high purity were used for the packaging of rAAV viral particles. The successfully transfected cells with AAV.eGFP emitted green fluorescent light. The transfection efficiency was determined by counting fluorescent cells and total cells as indicated by the following equation:

Transfection Efficiency (%)=Number of fluorescent cells/Total cells×100%

The fluorescent images at 72 h post-transfection indicated more than 90% efficiency. Similar transfection efficiency of AAV.hsFlt1 and AAV.Ori were reproduced by using the same transfection protocol.

**Figure 1:**
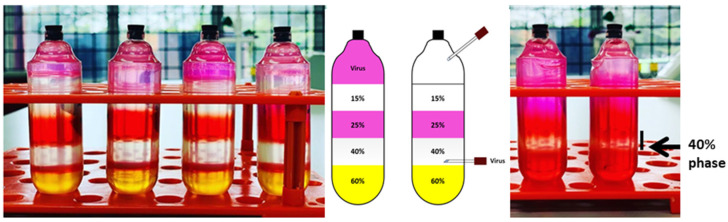
Iodixanol gradient for purification of rAAV before ultracentrifugation (**left**) and after ultracentrifugation (**right**). The black arrow indicates the layer containing purified recombinant virus particles.

The iodixanol gradient was used to purify viral particles because the iodixanol solution is inert, and thus, no toxicity is posed to cells. The purification steps are illustrated in Figure 1. The viral particles sedimented at 40% phase, and any proteinaceous materials near 40–25% phase were avoided. Around 3 to 4 mL of purified rAAV was collected using a 21-G needle with the bevel facing upward and attached to a syringe to ease the collection. The process was followed by concentrating and desalting on the next day. Titration was performed using qPCR with a standard curve for every new batch of recombinant virus particles produced. The final titration of a small batch of concentrated rAAV generated 10^5^ to 10^6^ copies/μL using 10 plates of 10-cm culture dish; whereas a large batch using 30 plates of 15-cm culture dish yielded more than 10^8^ copies/μL.

### 3.3. Expression of hsFlt-1 in MSCs

**Figure 2:**
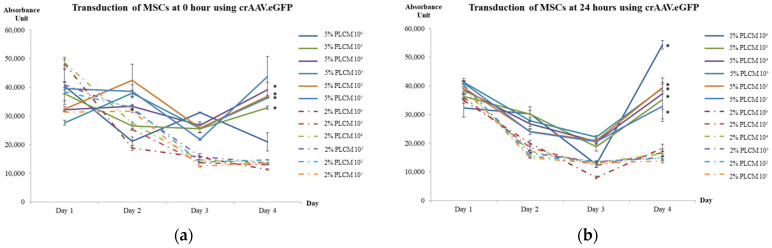
Optimization of MOI and various culture conditions by using rAAV.eGFP at (**a**) 0 h and (**b**) 24 h. Solid lines represented study groups cultured in 5% PLCM; Dotted lines represented study groups cultured in 2% PLCM. Data are expressed as mean (±SEM) with * p < 0.005 in comparison with 2% and 5% PLCM on Day 4.

**Figure 3:**
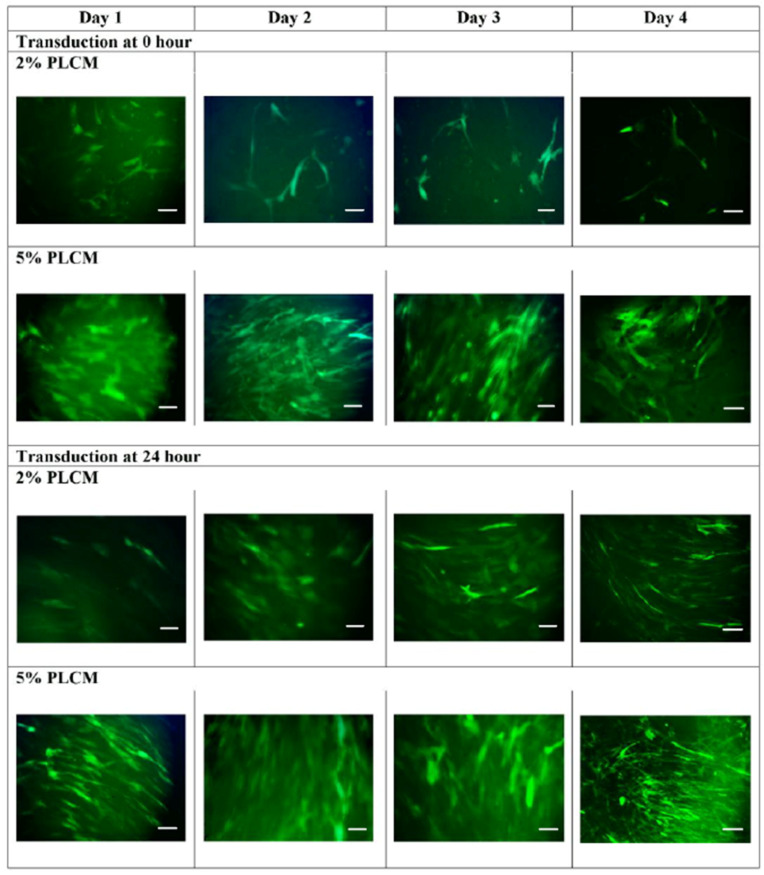
Transduction of MSCs using rAAV.eGFP at 0 h and 24 h cultured with 2% PLCM and 5% PLCM. The green fluorescent images were photographed on days 1, 2, 3, and 4 with an MOI of 1 × 10^6^ (magnification = 20×; scale bar = 150 µm).

**Figure 4:**
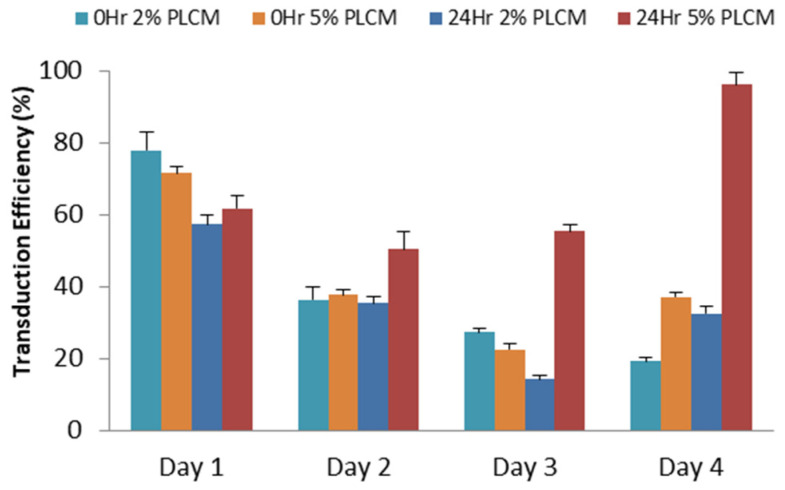
The relative transduction efficiency of eGFP gene expression in MSCs was evaluated under different serum concentrations and at various time points after transduction in MOI of 1 × 10^6^. Data are expressed as the mean (±SEM) over a 4-day period for comparison.

The effects of MOI and various culture conditions, including the percentage of complete media serum and attachment of MSCs, were optimized in a single experiment. The green fluorescent protein signal emissions of each study group were measured daily for four days using a spectrophotometer. The study group cultured in 5% serum transduced right after plating at 0 h (Figure 2a) showed a significant increase in fluorescent signals on Day 3 and Day 4 (One-Way ANOVA test; *p* < 0.005). A post-hoc analysis using Student’s *T*-Test (* p < 0.05) further validated on Day 4 demonstrated that all MOIs, except the MOI of 1 × 10^1^ and 1 × 10^6^, were significantly increased in green fluorescent signals. Similarly, Figure 2b demonstrated a significant downward trend in the 2% serum culture group on Day 2 onwards when the cells were transduced after being plated for 24 h (One-Way ANOVA test; *p* < 0.05). The same post-hoc analysis validated that the higher fluorescent signals emitted from the 5% serum culture group were significant across all MOIs on Day 4, except the MOI of 1 × 10^5^ (Student’s *T*-Test; * p < 0.05). The results suggested the serum culture group of 5% exhibited higher transduction efficiency for cells plated at 0 h and 24 h. Further comparison was made between 0 h and 24 h for the 5% serum group, demonstrating that MOI of 1 × 10^6^ emitted the highest fluorescent signals on day 4. The findings were further supported by the microscopic fluorescent images, as shown in Figure 3. The green fluorescent protein was observed to be highly expressed in MOI of 1 × 10^6^, especially in 5% PLCM cultured groups. There was no obvious signal observed in other MOI groups. The relative transduction efficiency of eGFP gene expression in MSCs was evaluated under different serum concentrations, 2% and 5%, and at 0 and 24 h. The quantitative analysis was performed over a 4-day period for comparison (One-Way ANOVA test; *p* < 0.05). The study group, in which cells were plated at 24 h using 5% serum, exhibited the highest transduction efficiency, increasing from 50.36% on day 2 to 96.30% on day 4, compared to other groups (Figure 4). In contrast, the both groups plated at 0 h decreased sharply after day 1, from an average of 71.65 ± 3.63% on day 1 to 28.26 ± 4.95% on day 4.

### 3.4. Secretion of hsFlt-1 in MSCs

**Figure 5:**
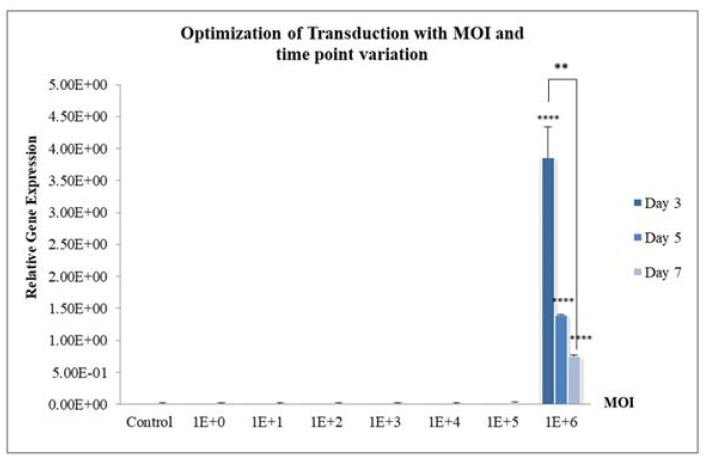
Quantification of hsFlt1 by qPCR with a range of MOIs and time points at Day 3, 5, and 7. Control indicates MSCs without transduction. Data are expressed as mean (±SEM) with ** *p* < 0.05 in comparison within the study group of 1 × 10^6^ MOI and **** *p* < 0.0001 in comparison with other MOI study groups.

To confirm the expression from various MOIs on transduced MSCs, a gene expression study using qPCR was performed by collecting the transduced cells on day 3, 5, and 7. The result as shown in Figure 5 indicates only MOI of 1 × 10^6^ significantly upregulated the hsFlt1 gene, especially highest on Day 3 in comparison with day 5 and 7 (One-Way ANOVA test; ** *p* < 0.05). The gene expressions were highly significant for the study group with the MOI of 1 × 10^6^ compared to other MOIs (One-Way ANOVA test; **** p < 0.0001). These quantitative results supported the findings shown on microscopic images in Figure 3. At the gene level, MSCs transduced at the MOI of 1 × 10^6^ revealed a prominent expression in the targeted hsFlt1 gene. This shows successful production of high-efficiency transduced MSCs. The MOI of 1 × 10^6^ was used in the subsequent study for functionality assays.

Successful translation of genes to produce functional molecules in the form of proteins is crucial to studying the effects of genetically modified cellular therapy. The subsequent study for translation of mRNA to protein is necessary to investigate the functionality of transduced MSCs in angiogenesis study. Therefore, ELISA was performed to study the hsFlt-1 protein expression on transduced MSCs (MSCs.hsFlt1).

**Figure 6:**
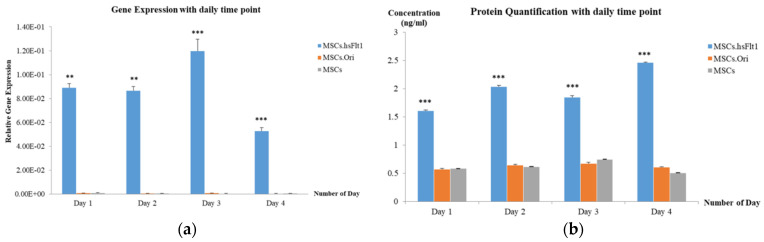
Quantification for secretion of MSCs.hsFlt1. (**a**) Gene expression of hsFlt-1 by qPCR with MOI of 1 × 10^6^ and time point from Day 0 to Day 4 was studied. MSCs represented control without transduction. Data are expressed as mean (± SEM) with ** *p* < 0.05 and *** *p* < 0.001 compared to MSCs.Ori and MSCs. (**b**) Protein quantification of hsFlt1 by ELISA with MOI of 1 × 10^6^ and time points from Day 0 to Day 4 was studied. Data are expressed as mean (± SEM) with *** *p* < 0.001 compared to MSCs.Ori and MSCs.

Figure 5 shows the highest gene expression was recorded on Day 3 compared to Day 5 and Day 7. The experiment was repeated from Day 0 to Day 4 to confirm the day with the highest expression. Consistent with the findings, Figure 6a revealed that MSCs.hsFlt1 on Day 3 demonstrated the highest gene expressions with significant effect compared to other study groups. In the same experiment, conditioned media were assayed each day for ELISA. The transduced MSCs with targeted hsFlt-1 gene revealed the highest protein expressions on Day 4, as demonstrated in Figure 6b. There were significant effects (One-Way ANOVA test; *** p < 0.001) on each day for MSCs.hsFlt1 in comparison to MSCs.Ori and MSCs without modification. In contrast, the protein expression showed a reverse trend compared to gene expression on Day 3 and Day 4. This suggests that the genetically modified MSCs with the lowest overexpression of hsFlt-1 gene products produced the highest level of functional anti-angiogenesis proteins.

### 3.5. Inhibition of Tube Formation with MSCs.hsFlt1

To simulate the in vivo microenvironment for the next phase of the study, a tube formation assay was performed to examine the formation of capillary-like structures in a three-dimensional matrigel. The co-culture of transduced MSCs and HUVECs in the basement matrigel was initiated on Day 3, which was a day before the highest hsFlt-1 secretions, based on Figure 6. Bevacizumab, as the current standard of treatment for corneal neovascularization, served as the positive control in this study.

**Figure 7:**
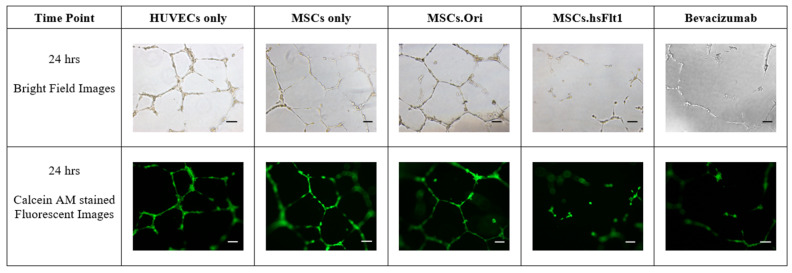
Microscopic images of the tube formation assay by forming HUVEC tube networks in basement matrigel in a 24-well plate. Control groups, only MSCs and transduced MSCs were seeded in transwell, respectively, and co-cultured with HUVECs in the basement matrigel. Fluorescent images were the outcome of the Calcein AM reagent. All microscopic images were captured at the magnification of 10× (scale bar = 10 µm).

At the end of the co-culture experiment, the microscopic images were photographed, as presented in Figure 7. All the microscopic images were quantified and the metrics, including total tube lengths, total loops, total branching points, and mean loop area, were analyzed and compared to the control group, HUVEC.

**Figure 8:**
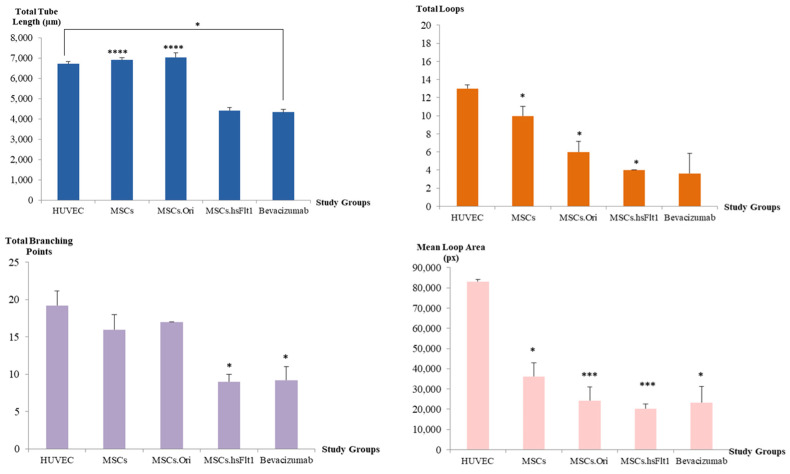
Quantitative analysis of the tube formation assay was evaluated using total tube length, total loops, total branching points, and mean loop area from each microscopic image. Data are expressed as mean (±SEM) with * *p* < 0.05, *** *p* < 0.001, and **** *p* < 0.0001 compared to the HUVEC control group.

The quantitative results plotted in Figure 8 were consistent with the microscopic images in Figure 7. All the study groups exhibited similar effects in comparison with the qualitative images and quantitative results on total tube lengths, total loops, total branching points, and mean loop area, where, MSCs.hsFlt1 showed significant effects in inhibiting the growth of HUVECs (Student’s *T*-Test; * *p* < 0.05, *** *p* < 0.001 and **** *p* < 0.0001). A significant reduction of 31.8% to 35% in total tube lengths was identified in MSCs.hsFlt1 comparable to other groups except for bevacizumab. In comparison to the current standard of care using bevacizumab, both treatments showed a similar anti-angiogenesis effect, as indicated by a decrease in the vessel networks. However, the concentration used in the MSCs.hsFlt1 study group was at 12.3 ng/mL compared to 50 µg/mL of bevacizumab, which was 4065-fold lower than the bevacizumab control group.

## 4. Discussion

This in vitro study has demonstrated a potential breakthrough in the treatment of neovascularization as an anti-angiogenic gene therapy. The capability of MSCs to bypass immune recognition has important implications for their potential use in therapeutic applications. Stromal cell therapies provide an alternative to treat a wide range of diseases. Still, the success of these treatments may depend on the ability of the transplanted cells to survive and function in the recipient’s body without being attacked by the immune system [18]. MSCs have proved to have immune-modulating effects in vitro and in vivo [18,19,20]. These immune-privileged properties of MSCs contributed to the successful rate of autologous and allogeneic transplantations.

However, genetically engineered stromal cells by introducing genes, especially in primary stromal cells, can be relatively challenging. Unlike established immortal cell lines, primary stromal cells are difficult to transduce due to their natural quiescent state and slow cell proliferation rate [21]. Quiescence is critical for retaining stemness and regenerative capacity [22]. Yet, this unique stromal cell property may be one of the contributing factors to poor viral integration during transduction. Furthermore, primary stromal cells often have low transduction efficiencies in experiments [23,24]. These difficulties in transducing primary stromal cells have led researchers to develop novel gene delivery methods and optimize existing techniques to improve transduction efficiency and maintain the high viability of the cells.

Stromal cells, especially from primary sources, are known to be difficult to transduce, yet the data for transduction efficiency on human MSCs is lacking [25,26,27]. Song et al. tested 10 serotypes of AAV, but none successfully transduced human hematopoietic stem cells in vitro [23]. Ellis et al. investigated 34 types of mammalian primary cells and cell lines transduced using 10 different AAV serotypes [28]. The authors reported that MSCs were not efficiently transduced in all the serotypes and only 0.2% efficiency in AAV2 using an MOI of 1 × 10^5^. Therefore, a higher MOI is needed to transduce this challenging cell type effectively.

Previous studies indicated that serum plays a crucial role in supporting the growth of MSCs [29,30]. The present observation is consistent with the findings where the normal percentage of serum, 5%, showed a higher survival rate in the process of transduction. MSCs transduced in 5% serum culture medium and plated at 24 h had the highest cell viability rate, even in an MOI of 1 × 10^6^. This result is supported by the emission of the highest fluorescent signals and quantified transduction efficiency of 96.30 ± 1.18% observed in the same MOI study group on day 4.

Furthermore, several studies demonstrated that high MOI is needed to achieve higher transduction efficiency in stem cells [31,32]. Asuri et al. reported using as high as MOI of 1 × 10^5^ to achieve an efficiency of 48.21 ± 12.92% in human pluripotent stem cells [33]. Kang et al. documented that AAV2 using an MOI of 1 × 10^5^ produced the highest transgenic expression in human adipose-derived MSCs [34]. Kim et al. further demonstrated bone marrow-derived and umbilical cord blood-derived MSCs were effectively transduced at an MOI of 1 × 10^4^ in a healthy state with no detection of cytotoxicity, and more than 90% of transduction efficiency was achieved [35]. These findings further validated the results in the current study that a high MOI of 1 × 10^6^ was needed to produce a high level of transgenic expression in human MSCs. As there is limited to no data on transgenic expression using human MSCs isolated from the umbilical cord, the results in the present study may contribute to the database collection of different primary human stromal cell sources using the viral transduction method.

Generally, the key assumption for gene and protein expressions is that they are correlated, as mRNA must be translated to produce a protein. The step to translate mRNA requires the transfer of RNA to work together for protein synthesis [36]. However, the lack of correlation between the level of mRNA and protein may be due to the complexity of gene transcription and translation processes. Gilbert S. F. and Greenbaum et al. summarized the possible shortfall in protein secretion levels for three possible reasons [37,38]. First, the persistence of differential mRNA plays a role in the process. The longer the half-life of an mRNA, the more proteins can be translated. Previous studies showed that mRNA was proven to be selectively stabilized under certain circumstances by factors such as time and cell types [39,40]. Thus, a preliminary study was essential to investigate the mRNA short-life of transduced MSCs. Second, the control of mRNA translation was regulated by cytoplasmic localization; and third, the protein levels were jointly defined by protein half-lives, translation rate constants, and mRNA levels [41]. A recent study stated that the levels of transcripts and proteins do not correlate well unless a gene-specific RNA-to-protein conversion factor independent of the tissue type is studied [42].

Gene transcription and translation are complex processes; the total amount of proteins to be translated can be regulated by the time of messenger RNA (mRNA) translation [38]. The optimum level of protein secretions on a particular day is crucial to evaluate anti-angiogenesis effects. Therefore, the findings are explanatory and sensible for both protein and gene expressions with low correlation. Since protein is the functional molecule of gene product, the highest level of protein secreted on Day 4 was used to evaluate the inhibition of the tube formation assay.

The tube formation assay is a functional study for regulating cell signaling pathways in angiogenesis. Therefore, the introduction of anti-angiogenesis inhibitors may clarify the rational therapeutic proposition in corneal neovascularization in vitro. The finding showed the angiogenesis effect was neutralized by overexpression of the hsFlt-1 gene, resulting in an overall decrease in total tube length, total loops, total branching points, and mean loop area. The anti-angiogenic effects reduce overall network complexity by disrupting endothelial tube formation, leading to low metrics as indicated by the findings. This significant effect in growth inhibition by MSCs.hsFlt1 in vitro indeed exhibited a very prominent anti-angiogenic effect, which was achieved especially using a much smaller dosage. The current standard treatment with frequent bevacizumab injections has posed challenges for both patients and healthcare providers due to the inconvenience of frequent visits to healthcare facilities. Our findings successfully demonstrate that genetically modified MSCs overexpressing a low dosage of 12.3 ng/mL hsFlt-1 can achieve a comparable anti-angiogenic effect to bevacizumab.

These results present a promising alternative therapy for patients suffering from abnormal blood vessel growth, particularly in ocular conditions. Future studies suggest focusing on the evaluation of genetically modified MSCs’ therapeutic efficacy and safety using animal models. To study ocular conditions with safety and efficacy profiles, the rabbit model could be a suitable in vivo model to study corneal neovascularization [43,44]. The ocular injury can be induced using either chemical burn or mechanical injury prior to anti-angiogenesis cell-based treatment [45]. The previous studies demonstrated that treatments using genetically modified MSCs with AAV prove the expression of the introduced gene is often transient. A single dose of transiently modified MSCs might provide a one-time therapeutic effect, which could be ideal for certain indications, such as reducing acute inflammation or preventing the early stages of organ rejection, without the need for repeated treatments, and postulating that the immunogenicity risk will be relatively lower. Furthermore, utilizing MSCs as a gene delivery vehicle enables tissue repair to occur directly at the inflammatory site, enhancing the therapeutic potential of this cellular treatment. Moreover, the immune-modulating properties exhibited in MSCs allow for the preparation of this treatment as an off-the-shelf product, reducing the need for patient-specific cell preparations.

Thus, we proposed that future studies can focus on designing the approach of single-dose treatments with AAV-modified MSCs, and the transient gene expression could mitigate concerns about long-term immunosuppression properties raised by MSCs and immunogenicity risk by AAV viral vector integration. This strategy is particularly advantageous since the therapeutic effects would be short-lived yet sufficient to address conditions related to ophthalmology like corneal neovascularization. Consequently, this approach could make MSCs a safer option by reducing the risks of prolonged immune-related complications.

## 5. Conclusions

In conclusion, this in vitro study showcases an encouraging anti-angiogenesis effect on HUVECs using genetically modified MSCs with significantly lower dosage equivalence of hsFlt1 compared to the current standard treatment with bevacizumab. The optimized high-efficiency transduction protocol serves as a crucial step towards the scale-up of transduced MSCs for potential in vivo and clinical studies. These promising findings have the potential to revolutionize the treatment landscape for neovascularization, offering a more convenient, effective, and patient-friendly therapeutic option for individuals with angiogenic-related complications.

## Figures and Tables

**Table 1:** List of primer sequences and product sizes for qPCR analysis.

Gene	Primer Sequence 5′–3′
Glyceraldehyde 3-phosphate dehydrogenase (GADPH)	F: 5′-TCC CTG AGC TGA ACG GGA AG-3′ R: 5′-GGA GGA GTG GGT GTC GTC GCT GT-3′
Human Fms-related tyrosine kinase 1 (hFLT1(s7))	F: 5′-CCA TCA GCA GTT CCA CCA CT-3′ R: 5′-ACA CAG AGC CCT TCT GGT TG-3′

## Data Availability

The original contributions presented in this study are included in the article. Further inquiries can be directed to the corresponding author.

## References

[1] Phinney D.G. (2002). Building a consensus regarding the nature and origin of mesenchymal stem cells. J. Cell. Biochem..

[2] Choi S.H., Tamura K., Khajuria R.K., Bhere D., Nesterenko I., Lawler J., Shah K. (2015). Antiangiogenic variant of TSP-1 targets tumor cells in glioblastomas. Mol. Ther..

[3] Ewa Choy Y.W., Choy K.W., Woon K.S., Wafi M.A., Then K.Y., Then K.L. (2024). Genetically Engineered Mesenchymal Stem Cells Using Viral Vectors: A New Frontier in Anti-Angiogenic Therapy. Sains Malays..

[4] Wen Q., Jin D., Zhou C.-Y., Zhou M.-Q., Luo W., Ma L. (2012). HGF-transgenic MSCs can improve the effects of tissue self-repair in a rabbit model of traumatic osteonecrosis of the femoral head. PLoS ONE.

[5] Wang Q., Zhang Z., Ding T., Chen Z., Zhang T. (2013). Mesenchymal stem cells overexpressing PEDF decrease the angiogenesis of gliomas. Biosci. Rep..

[6] Obtulowicz P., Lech W., Strojek L., Sarnowska A., Domanska-Janik K. (2016). Induction of endothelial phenotype from wharton’s jelly-derived MSCs and comparison of their vasoprotective and neuroprotective potential with primary WJ-MSCs in CA1 hippocampal region ex vivo. Cell Transplant..

[7] Porada C.D., Almeida-Porada G. (2010). Mesenchymal stem cells as therapeutics and vehicles for gene and drug delivery. Adv. Drug Deliv. Rev..

[8] Choudhery M.S., Badowski M., Muise A., Harris D.T. (2013). Comparison of human mesenchymal stem cells derived from adipose and cord tissue. Cytotherapy.

[9] Guerra-Crespo M., Charli J.-L., Rosales-García V.H., Pedraza-Alva G., Pérez-Martínez L. (2003). Polyethylenimine improves the transfection efficiency of primary cultures of post-mitotic rat fetal hypothalamic neurons. J. Neurosci. Methods.

[10] Park H.-J., Yang F., Cho S.-W. (2012). Nonviral delivery of genetic medicine for therapeutic angiogenesis. Adv. Drug Deliv. Rev..

[11] Basner-Tschakarjan E., Mingozzi F. (2014). Cell-Mediated Immunity to AAV Vectors, Evolving Concepts and Potential Solutions. Front. Immunol..

[12] Santiago-Ortiz J.L., Schaffer D.V. (2016). Adeno-associated virus (AAV) vectors in cancer gene therapy. J. Control. Release.

[13] Hu M., Yang J.-L., Teng H., Jia Y.-Q., Wang R., Zhang X.-W., Wu Y., Luo Y., Chen X.-C., Zhang R. (2008). Anti-angiogenesis therapy based on the bone marrow-derived stromal cells genetically engineered to express sFlt-1 in mouse tumor model. BMC Cancer.

[14] Barleon B., Siemeister G., Martiny-Baron G., Weindel K., Herzog C., Marmé D. (1997). Vascular endothelial growth factor up-regulates its receptor fms-like tyrosine kinase 1 (FLT-1) and a soluble variant of FLT-1 in human vascular endothelial cells. Cancer Res..

[15] Terman B.I., Carrion M., Kovacs E., Rasmussen B., Eddy R., Shows T. (1991). Identification of a new endothelial cell growth factor receptor tyrosine kinase. Oncogene.

[16] Crosson S.M., Dib P., Smith J.K., Zolotukhin S. (2018). Helper-free Production of Laboratory Grade AAV and Purification by Iodixanol Density Gradient Centrifugation. Mol. Ther. Methods Clin. Dev..

[17] Chua K., Aminuddin B., Fuzina N., Ruszymah B. (2005). Insulin-transferrin-selenium prevent human chondrocyte dedifferentiation and promote the formation of high quality tissue engineered human hyaline cartilage. Eur. Cells Mater..

[18] Singh A.K., McGuirk J.P. (2016). Allogeneic Stem Cell Transplantation: A Historical and Scientific Overview. Cancer Res..

[19] Mao F., Xu W.-R., Qian H., Zhu W., Yan Y.-M., Shao Q.-X., Xu H.-X. (2010). Immunosuppressive effects of mesenchymal stem cells in collagen-induced mouse arthritis. Inflamm. Res..

[20] Ghannam S., Bouffi C., Djouad F., Jorgensen C., Noël D. (2010). Immunosuppression by mesenchymal stem cells: Mechanisms and clinical applications. Stem Cell Res. Ther..

[21] Nolta J.A., Kohn D.B., Potten C.S. (1997). 15—Haematopoietic stem cells for gene therapy. Stem Cells.

[22] Urbán N., Cheung T.H. (2021). Stem cell quiescence: The challenging path to activation. Development.

[23] Song L., Kauss M.A., Kopin E., Chandra M., Ul-Hasan T., Miller E., Jayandharan G.R., Rivers A.E., Aslanidi G.V., Ling C. (2013). Optimizing the transduction efficiency of capsid-modified AAV6 serotype vectors in primary human hematopoietic stem cells in vitro and in a xenograft mouse model in vivo. Cytotherapy.

[24] Ling C., Bhukhai K., Yin Z., Tan M., Yoder M.C., Leboulch P., Payen E., Srivastava A. (2016). High-Efficiency Transduction of Primary Human Hematopoietic Stem/Progenitor Cells by AAV6 Vectors: Strategies for Overcoming Donor-Variation and Implications in Genome Editing. Sci. Rep..

[25] Canté-Barrett K., Mendes R.D., Smits W.K., van Helsdingen-van Wijk Y.M., Pieters R., Meijerink J.P.P. (2016). Lentiviral gene transfer into human and murine hematopoietic stem cells: Size matters. BMC Res. Notes.

[26] Ricks D.M., Kutner R., Zhang X.-Y., Welsh D.A., Reiser J. (2008). Optimized Lentiviral Transduction of Mouse Bone Marrow-Derived Mesenchymal Stem Cells. Stem Cells Dev..

[27] Lin P., Correa D., Lin Y., Caplan A.I. (2011). Polybrene Inhibits Human Mesenchymal Stem Cell Proliferation during Lentiviral Transduction. PLoS ONE.

[28] Ellis B.L., Hirsch M.L., Barker J.C., Connelly J.P., Steininger R.J., Porteus M.H. (2013). A survey of ex vivo/in vitro transduction efficiency of mammalian primary cells and cell lines with Nine natural adeno-associated virus (AAV1-9) and one engineered adeno-associated virus serotype. Virol. J..

[29] Pal R., Hanwate M., Jan M., Totey S. (2009). Phenotypic and functional comparison of optimum culture conditions for upscaling of bone marrow-derived mesenchymal stem cells. J. Tissue Eng. Regen. Med..

[30] Potier E., Ferreira E., Meunier A., Sedel L., Logeart-Avramoglou D., Petite H. (2007). Prolonged hypoxia concomitant with serum deprivation induces massive human mesenchymal stem cell death. Tissue Eng..

[31] Kamel S.H. (2014). Adeno-Associated Virus (AAV) Transduction of Primary Human CD4+T Lymphocytes. Master’s Thesis.

[32] McMahon J.M., Conroy S., Lyons M., Greiser U., O’Shea C., Strappe P., Howard L., Murphy M., Barry F., O’Brien T. (2006). Gene Transfer into Rat Mesenchymal Stem Cells: A Comparative Study of Viral and Nonviral Vectors. Stem Cells Dev..

[33] Asuri P., Bartel M.A., Vazin T., Jang J.-H., Wong T.B., Schaffer D.V. (2012). Directed Evolution of Adeno-associated Virus for Enhanced Gene Delivery and Gene Targeting in Human Pluripotent Stem Cells. Mol. Ther..

[34] Kang Y., Liao W.-M., Yuan Z.-H., Sheng P.-Y., Zhang L.-J., Yuan X.-W., Lei L. (2007). In vitro and in vivo induction of bone formation based on adeno-associated virus-mediated BMP-7 gene therapy using human adipose-derived mesenchymal stem cells. Acta Pharmacol. Sin..

[35] Kim S.J., Lee W.I., Heo H., Shin O., Kwon Y.K., Lee H. (2007). Stable gene expression by self-complementary adeno-associated viruses in human MSCs. Biochem. Biophys. Res. Commun..

[36] Agirrezabala X., Liao H.Y., Schreiner E., Fu J., Ortiz-Meoz R.F., Schulten K., Green R., Frank J. (2012). Structural characterization of mRNA-tRNA translocation intermediates. Proc. Natl. Acad. Sci. USA.

[37] Greenbaum D., Colangelo C., Williams K., Gerstein M. (2003). Comparing protein abundance and mRNA expression levels on a genomic scale. Genome Biol..

[38] Gilbert S.F. (2000). Control of Gene Expression at the Level of Translation. Developmental Biology.

[39] Guyette W.A., Matusik R.J., Rosen J.M. (1979). Prolactin-mediated transcriptional and post-transcriptional control of casein gene expression. Cell.

[40] Decker C.J., Parker R. (1994). Mechanisms of mRNA degradation in eukaryotes. Trends Biochem. Sci..

[41] Buccitelli C., Selbach M. (2020). mRNAs, proteins and the emerging principles of gene expression control. Nat. Rev. Genet..

[42] Edfors F., Danielsson F., Hallström B.M., Käll L., Lundberg E., Pontén F., Forsström B., Uhlén M. (2016). Gene-specific correlation of RNA and protein levels in human cells and tissues. Mol. Syst. Biol..

[43] Zernii E.Y., Baksheeva V.E., Iomdina E.N., Averina O.A., Permyakov S.E., Philippov P.P., Zamyatnin A.A., Senin I.I. (2016). Rabbit models of ocular diseases: New relevance for classical approaches. CNS Neurol. Disord.-Drug Targets.

[44] Del Amo E.M., Urtti A. (2015). Rabbit as an animal model for intravitreal pharmacokinetics: Clinical predictability and quality of the published data. Exp. Eye Res..

[45] Gupta S., Martin L.M., Zhang E., Sinha P.R., Landreneau J., Sinha N.R., Hesemann N.P., Mohan R.R. (2023). Toxicological effects of ocular acrolein exposure to eyelids in rabbits in vivo. Exp. Eye Res..

